# Marking the Profile of the Microflora of the Endometrium and Uterine Cervix in Women as a Potential Factor Determining the Effectiveness of In Vitro Fertilization

**DOI:** 10.3390/jcm11123348

**Published:** 2022-06-10

**Authors:** Anna Bednarska-Czerwińska, Michał Czerwiński, Emilia Morawiec, Aleksandra Łach, Anna Ziaja, Adrian Kusaj, Patrycja Strączyńska, Dorota Sagan, Dariusz Boroń, Beniamin Oskar Grabarek

**Affiliations:** 1 Gyncentrum Fertility Clinic, 40-121 Katowice, Poland; michalczerwinski1992@gmail.com (M.C.); emilia.wojdas@gmail.com (E.M.); straczynska.patrycja@gmail.com (P.S.); bgrabarek7@gmail.com (B.O.G.); 2 Faculty of Medicine, University of Technology, Academy of Silesia in Katowice, 41-800 Zabrze, Poland; 3 American Medical Clinic, 40-600 Katowice, Poland; 4 Department of Histology, Cytophysiology and Embryology, Faculty of Medicine, University of Technology, Academy of Silesia in Katowice, 41-800 Zabrze, Poland; aleksandra.lach.12@interia.pl (A.Ł.); anna.ziaja99@gmail.com (A.Z.); adrian1040@onet.pl (A.K.); dariusz@boron.pl (D.B.); 5 Department of Microbiology, Faculty of Medicine, University of Technology, Academy of Silesia in Katowice, 41-800 Zabrze, Poland; 6 Department of Gynecology, Obstetrics and Oncological Gynecology in Bytom, Medical University of Silesia in Katowice, 41-902 Bytom, Poland; 7 Medical Center Dormed Medical SPA, 28-105 Busko-Zdroj, Poland; dorotasagan@gmail.com; 8 Department of Gynaecology and Obstetrics, Faculty of Medicine, University of Technology, Academy of Silesia in Katowice, 41-800 Zabrze, Poland; 9 Department of Gynecology and Obstetrics with Gynecologic Oncology, Ludwik Rydygier Memorial Specialized Hospital, 31-826 Kraków, Poland; 10 Department of Gynecology and Obstetrics, TOMMED Specjalisci od Zdrowia, 40-662 Katowice, Poland

**Keywords:** microbiome, endometrium, microbiota, uterine cervix, in vitro fertilization, next-generation sequencing

## Abstract

One promising research trend involves evaluating the influence of microbiota in the reproductive system of women on becoming pregnant and maintaining pregnancy. The goal of this study was to define the microflora profile of the endometrium and uterine cervix in women qualified for an in vitro fertilization (IVF) procedure, which is expected to contribute to increasing the percentage of successful IVF implantations. Based on the conducted molecular analysis in the collected swabs, 22 bacterial strains were identified. Eleven strains (57%) that were isolated belong to the physiological microflora, the most common strain of which was Lactobacillus. Eight of the isolated strains (33%) were pathological microflora, among which the most common bacteria were from the Enterobacteriaceae family (which includes *E. coli*, Shigella, and Salmonella). Finally, three of the bacterial strains (10%) may be a component of both physiological or pathological microflora of the vagina: *Bifidobacterium breve*, *Bifidobacterium longum* group, and *Alloscardovia omnicolens*. The presence of *Escherichia coli* was detected in six women, *Staphylococcus aureus* also in six patients, *Atopobium parvulum* in three, *Streptococcus salivarius* group in three, *Enterococcus faecalis* in four, and *Aerococcus christensenii* in two patients. We found statistically significant relationships (*p* < 0.05) between *Lactobacillus fermentum* and *Enterococcus faecalis*, *Lactobacillus delbrueckii* and *Escherichia coli* groups, *Lactobacillus FN667084_s* and *Staphylococcus aureus* groups, as well as *Lactobacillus fermentum* and *Streptococcus agalactiae*. Based on the conducted study, it may be confirmed that the endometrium is, to a large extent, colonized by lactic acid bacilli. Apart from that, endometrial dysbiosis was not noted in patients qualified for the IVF procedure.

## 1. Introduction

Many women struggle with the problem of infertility, the cause of which varies for each woman. The World Health Organization has reported that infertility is a social disability. At present, many couples do not want to have children (e.g., due to wanting to concentrate on their professional careers), and if they indeed decide to have offspring, they want to do so within a short period of time, which is accompanied by a high level of stress [1]. There is a conviction that problems associated with fertility can be a stress-inducing factors. The inability to be a mother causes emotional problems for women, as well as anxiety, which greatly hinders becoming pregnant and maintaining pregnancy. The inability to conceive a child is, naturally, a serious problem when it comes to the woman’s psyche, and, in many cases, having a child is a sign of personal fulfillment [2,3,4]. Moving away from the social and mental aspects of infertility, it is important to point out that the physical health of a woman is key in the fertilization process. The most common reasons for infertility are endometriosis, polycystic ovary syndrome (POCS), as well as Fallopian tube blockage, which is often a result of inflammation of the reproductive organs. The uterine factor and ovulatory dysfunctions are also of paramount importance [5,6]. At present, infertility is a disease of civilization. The problem of infertility affects approximately 10–16% of people during child-bearing age [7,8,9,10]. Assisted reproductive technologies (ART) are the basis of contemporary techniques for infertility treatment. Their effectiveness—given as a coefficient of successful pregnancies—is quite low, approximately 25–35% [11,12,13], and depends on additional procedures undertaken by the unit conducting the ART.

The physiological bacterial flora of the vagina makes up a complex environment that can be altered under the influence of numerous factors, such as the woman’s age, the use of medications and external substances which the mucosa may come in contact with, the woman’s sexual activity, and the hormonal status of the patient [14]. The uterine cervix is a site of direct passage of bacteria between the vagina and the uterus. It is widely known that the Lactobacillus bacteria, as a component of the vaginal microbiota, stabilize its microenvironment. Their presence has also been confirmed in the male reproductive system, the sperm, as well as in the female reproductive system [7].

In recent years, studies on the significance of changes in microbiota on fertility dysfunctions have been undertaken. The protective role of Lactobacillus bacteria is commonly known; however, the mechanism of their activity requires further, more detailed studies. It is possible that a low number of Lactobacillus may be connected with the inability to implant an embryo [15,16,17]. The microflora profile of the vagina is important for the preimplantation process and also influences the maintenance of the pregnancy. It is suggested that the changes occurring during pregnancy in the vaginal and intestinal microbiota of the mother may be of adaptive significance, enabling the child to draw energy more effectively from the mother’s blood [18]. It has recently been reported that, during pregnancy, the microbiome undergoes numerous changes, denoting an active reaction of the mother, most likely aimed at changing the condition of the immune system, as well as metabolic and immune adaptations [19].

One of the most promising trends in such studies involves the evaluation of the influence of the microbiota of a woman’s reproductive system on becoming pregnant and maintaining pregnancy [6,9,10]. Dysbiosis of the endometrium may be a likely cause of failure of in vitro fertilization (IVF). A dysfunctional composition of the microbiota may cause the incidence of a chronic inflammatory condition of the endometrium, including endometriosis, leading to reoccurring implantation failures [13,20,21]. It is important to note that a non-beneficial microbiological profile of the vagina often does not present any clinical symptoms. It has also been proven that women who have inappropriate vaginal microbiota composition exhibit a 1.4 times lower chance of becoming pregnant after the use of ARTs, in comparison with women who have normal microbiota composition. Studies have shown that as many as 19% of infertile women are diagnosed with bacterial vaginal infection. Bacterial vaginosis (BV) may negatively influence the clinical factor of pregnancies among women subject to IVF. It has been shown that only 9% of the women diagnosed with BV became pregnant after the use of ARTs. Attempts are currently being made to determine the appropriate composition of the microbiota of the uterus and evaluate its influence on fertility [9]. The goal of the present study was to determine the profile of the microflora of the endometrium and the uterine cervix in women qualified for the IVF procedure, which may contribute to an increase in the percentage of successful IVF implantations.

## 2. Materials and Methods

### 2.1. Subjects

The study included 142 patients, aged 26–45 years old, of child-bearing age, who were patients of the Gyncentrum Clinic (Poland), and who were qualified for the in vitro procedure. In this group of patients, 183 samples in total were tested. However, we obtained 11 negative results, due to the low quality of swabs and the small amount of microbiota being impossible to efficiently isolate. Based on the measured beta-human chorionic gonadotropin (beta hCG) concentration 14 days after embryo implantation, 44 women were pregnant.

In Table 1, the basal characteristics of the participants are provided.

In turn, the inclusion and exclusion criteria for this study are presented in Table 2.

### 2.2. Next-Generation Sequencing

Endometrial swabs were collected on the Swab Collection and DNA Preservation Tube Transport Medium (Norgen Biotech Corp., 3430 Schmon Pkwy, Thorold, ON L2V 4Y6, Canada). Isolation was performed with a NucleoSpin^®^ Tissue kit, Genomic DNA from tissue, modified with the use of glass beads NucleoSpin Bead Tubes Type B (Macherey-Nagel, Hirsackerstr. 7—Postfach 255, 4702 Oensingen, Switzerland). DNA isolates were subjected to qualitative and quantitative evaluation, and libraries were prepared based on the Illumina-16S Metagenomic Sequencing Library Preparation (16S Sequencing) protocol. Library indexing was performed using a Nexter XT Kit (Illumina, 5200 Illumina Way, San Diego, CA 92122, USA). Fragments were purified using the MagSi-NGS MagSi-NGS (Magtivio, Daelderweg 9, 6361 HK Nuth, The Netherlands), and their analysis was conducted using a Fragment Analyzer (Agilent, 5301 Stevens Creek Blvd Santa Clara, Santa Clara, CA 95051, USA). Library concentrations were measured with a QuantiFluor ONE dsDNA Kit (Promega Corporation 2800 Woods Hollow Road, Madison, WI 53711, USA) on the Quantus ™ Fluorometer (Promega Corporation 2800 Woods Hollow Road, Madison, WI 53711, USA) and normalized to 4 nM. Sequencing was performed on the Illumina Miseq 2 × 300 bp platform. Analysis of the obtained sequences was performed based on the resources of the EzBioCloud platform (EzBiome Inc., 704 Quince Orchard Rd, Gaithersburg, MD 20878, USA). The taxonomic identification of bacteria to the species level was carried out using the implemented Usearch algorithm. The validation of the results was performed based on the ZymoBIOMICS Microbial Community Standard (Zymo Research, 17062 Murphy Ave., Irvine, CA 92614, USA) microbiological controls.

### 2.3. Statistical Analysis

Statistical analysis was conducted using Statistica 13.3 PL software (Statsoft, Cracow, Poland), assuming a significance level (*p*) of <0.05. During the first stage, the Shapiro–Wilk test was conducted in order to determine the normality of distribution of the obtained results. Due to the fact that its assumptions were fulfilled, the statistical analysis was performed with the use of parametric methods: a one-way ANOVA test, preceded by the Levene test and followed by a post hoc Tukey test. The analysis of the dependence of the presence of individual micro-organisms was conducted with Pearson’s correlation coefficient. The analysis excluded the strains of bacteria that were present only in a single patient, although they are presented in the results. The results for the number of individual strains of bacteria are presented as an average ± standard deviation (x ± SD) and a 95% confidence interval was considered.

According to the data published by the Central Statistical Office (GSO) in 2021, of 19,763,000 women in Poland, 5,261,203 women are aged 18–45 [22]. The number of participants in the study was determined using the statistical tool. Assuming a *p*-value of <0.05 and a maximum error value estimated at 9%, the required number of respondents for the study was 119 [23].

### 2.4. Ethics

The study was conducted according to the guidelines of the Declaration of Helsinki and approved by the Institution of the Bioethical Committee operating at the Regional Medical Chamber in Krakow. No. 161/KBL/OIL/2021 were obtained for this study. Informed consent was obtained from all subjects involved in the study. Written informed consent was obtained from the patient(s) to publish this paper.

## 3. Results

### 3.1. Composition of the Endometrium Microflora

**Figure 1:**
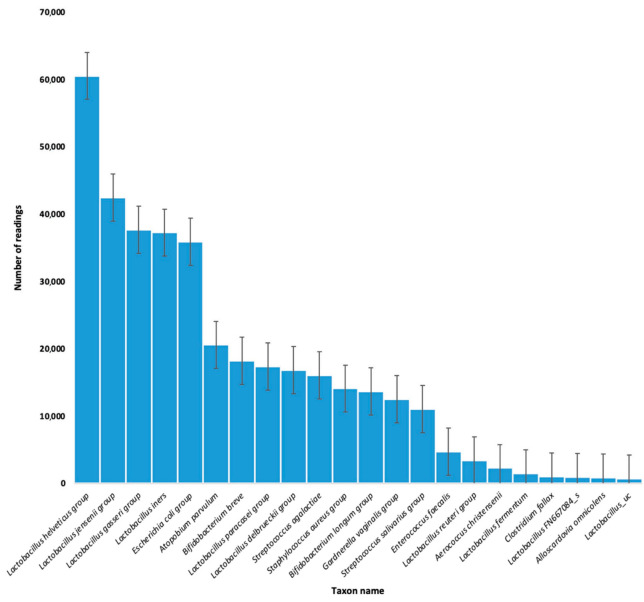
Number of readings of individual bacteria strains colonizing the endometrium and the uterine cervix of patients qualified for the IVF procedure.

**Figure 2:**
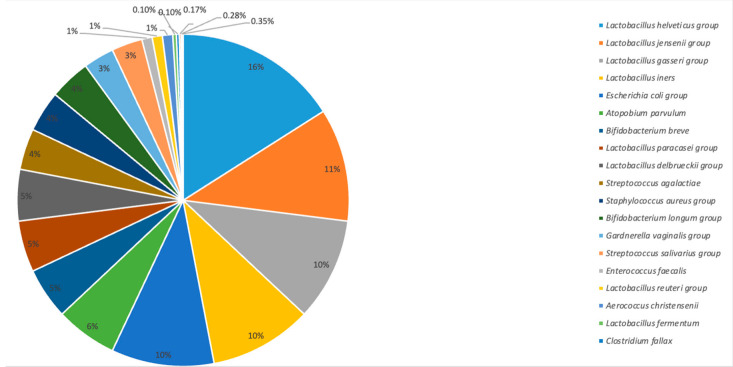
Percentage of individual bacteria strains colonizing the endometrium and the uterine cervix of patients qualified for the IVF procedure.

Based on the conducted molecular analysis, 22 strains of bacteria were identified in the collected swabs: 11 strains (57%) were part of the physiological microflora, the most numerous being Lactobacillus; 8 of the isolated strains (33%) were from the pathological microflora, the most numerous of which were bacteria from the Enterobacteriaceae family, which includes *E. coli*, *Shigella*, and *Salmonella*. On the other hand, three strains of bacteria (10%) may have been a component of both physiological or pathological microflora of the vagina, including Bifidobacterium breve, Bifidobacterium longum group, and Alloscardovia omnicolens. The presence of the Escherichia coli group was confirmed in six of the women, Staphylococcus aureus group also in six, while Aerococcus christensenii was confirmed in two patients (Table 3; Figure 1 and Figure 2).

Statistically significant differences in the number of individual strains were noted between the *Lactobacillus helveticus* group and *Aerococcus christensenii* (*p* = 0.018682); *Lactobacillus helveticus* group and *Bifidobacterium breve* (*p* = 0.000569); *Lactobacillus helveticus* group and *Enterococcus faecalis* (*p* = 0.044475); *Lactobacillus helveticus* group and *Gardnerella vaginalis* group (*p* = 0.000131); *Lactobacillus helveticus* group and *Lactobacillus fermentum* (*p* = 0.015366); *Lactobacillus helveticus* group and *Lactobacillus FN667084_s* (*p* = 0.013261); *Lactobacillus helveticus* group and *Lactobacillus gasseri* group (*p* = 0.004716); *Lactobacillus helveticus* group and *Lactobacillus reuteri* group (*p* = 0.000015); *Lactobacillus helveticus* group and *Lactobacillus paracasei* group (*p* = 0.003550); *Lactobacillus jensenii* group and *Lactobacillus reuteri* group (*p* = 0.000015); *Lactobacillus reuteri* group and *Lactobacillus gasseri* group (*p* = 0.010148); *Staphylococcus aureus* group and *Lactobacillus* *FN667084_s* (*p* = 0.003550); and *Lactobacillus jensenii* group and *Lactobacillus reuteri* group (*p* = 0.010148). The exact results of the post hoc test are presented in the Supplementary Materials (Table S1).

### 3.2. Incidence Dependences between Individual Isolated Bacteria Strains

Later, an evaluation was conducted regarding whether there existed statistically significant dependences (*p* < 0.05), which were noted between *Lactobacillus fermentum* and *Enterococcus faecalis* (r = −0.998903); *Lactobacillus delbrueckii* group and *Escherichia coli* group (r = −0.998903); *Lactobacillus delbrueckii* group and *Lactobacillus paracasei* group (r = −0.999946); *Lactobacillus fermentum* and *Streptococcus agalactiae* (r = 0.997254); *Bifidobacterium breve* and *Lactobacillus reuteri* group (r = −0.999918); *Lactobacillus gasseri* group and *Lactobacillus paracasei* group (r = 0.409367); *Lactobacillus helveticus* group and *Lactobacillus paracasei* group (r = 0.400625); *Lactobacillus jensenii* group and *Lactobacillus fermentum* (r = −0.999946); *Lactobacillus jensenii* group and *Lactobacillus helveticus* group (r = 0.409367); *Lactobacillus jensenii* group and *Lactobacillus iners* (r = 0.400625); *Staphylococcus aureus* group and *Lactobacillus FN667084_s* (r = 0.997254); and *Lactobacillus paracasei* group and *Bifidobacterium longum* group (r = −0.999918).

## 4. Discussion

Owing to the development of modern diagnostic methods based on molecular biology, we presently understand that the mucous membrane of the uterus is not a completely sterile environment. A significant breakthrough was the commencement of studies of the metagenome based on sequencing 16S rRNA of micro-organisms inhabiting the vagina and the uterus [24]. It is important to remember that a healthy microbiome of the vagina and endometrium, in comparison to the microbiome of the digestive system, is relatively unvaried and is mostly colonized by *Lactobacillus* [1,2]. Nevertheless, Miles et al. [25] and Winters et al. [26] have shown that, in endometrium samples obtained during hysterectomy, the dominant bacteria are *Acinetobacter*, *Pseudomonas*, and *Corynebacterium*. However, it must be remembered that the studies conducted by Miles et al. [25] were performed on a small, pilot group of 10 patients, out of which 4 were of post-menopausal age, and 1 in the perimenopausal period. Moreover, in 8 out of 10 patients, a hysterectomy was performed for oncological reasons, which made them incompatible in relation to the inclusion criteria adopted in our study [3]. Similarly, the study of Winters et al., was carried out in a group of only 25 patients [4]. The significance of studying the microbiota of the endometrium within the context of seeking potential causes of infertility and the effectiveness of in vitro fertilization has been confirmed through the observations made by Lull et al. [27] who, similarly to the presented results, showed that the *Lactobacillus* genus is the dominant bacteria inhabiting the endometrium in women of pre-menopausal age, which has great significance when it comes to the success of the IVF procedure [5,6]. A probable cause that makes the *Lactobacillus* bacteria so important for maintaining homeostasis, becoming pregnant, and maintaining pregnancy, as well as the success of the IVF procedure, is the fact that *Lactobacillus* produce lactic acid, which causes a reduction in the pH of the vagina [7]. Lactic acid is a histone deacetylase inhibitor, thus strengthening the expression of genes and DNA repair; as a result, infections are limited without the induction of the inflammatory process, thus increasing fertility and increasing the chance of becoming pregnant and maintaining pregnancy [28]. In addition, the anti-inflammatory role of the *Lactobacillus* spp. in the vaginal microenvironment and the endometrium may be connected to the fact that these bacteria contribute to the overexpression of anti-inflammatory cytokines such as Interleukin-1, as well as the secretion of antimicrobial peptides such as IgA and IgG antibodies, β-defensins, mucins, secretory leukocyte protease inhibitors (SLPI), and neutrophil gelatinase-associated lipocalins (NGAL) [8,10]. The observations of Carosso et al., who assessed whether controlled ovarian stimulation and progesterone luteal supplementation have an influence on the vaginal and endometrial microbiota of women undergoing IVF, are also interesting. The authors showed that the mentioned treatments significantly affected the vaginal and endometrium microflora, noting the percentage decrease in Lactobacillus bacilli with a simultaneous increase in the percentage of pathological microflora [29].

In turn, Moreno et al., assessed the association between the endometrial microflora and fertility and the success of IVF in a population of 342 infertile couples. They noted that endometrial colonization by micro-organisms such as Atopobium, Bifidobacterium, Chryseobacterium, Gardnerella, Hemophilus, Klebsiella, Neisseria, Staphylococcus, and Streptococcus was significantly associated with the failure of the IVF method. At the same time, they emphasized that the share of Lactobacillus in the microflora can serve as a prognostic indicator for the success of IVF. Thus, microwave analysis of the endometrium prior to embryo implantation is useful for determining the success of IVF [30].

However, as the observations of Sezer et al., have shown, in the event of endometrial or cervical dysbiosis, possible supplementation with probiotics or synbiotics containing Lactobacillus bacilli should be carried out carefully and monitored. Indeed, it has been suggested that too high a proportion of lactobacilli can impair the microbiota, potentially causing idiopathic infertility. These authors showed that too many groups of lactic acid bacteria were associated with a ninefold higher risk of infertility (76.9% vs. 26.9%; *p* < 0.001; mean lactobacilli/total bacterial mass 38.2 vs. 76.3; *p* = 0.001) [31].

Of course, it should not be forgotten that infertility is a problem for a couple, not just a woman or a man. Okwelogu et al., assessed the microbiome of infertile couples; that is, the sperm in men and endometrium in women. They observed that the ejaculate microbiome is more diverse than the endometrial microbiome. On the basis of the conducted tests, they sought to indicate the microbiota pattern in men and women, which is associated with the success of the IVF procedure. Indeed, they showed a different percentage of individual bacteria making up the ejaculate microbiome in men, including normospermia, oligospermia, azoospermia, asthenozoospermia, teratozoospermia, oligoasthenozoospermia, and pyospermia. They also found that the most statistically successful IVF procedure was in couples where *Lactobacillus jensenii* and Faecalibacterium dominated the semen of men, and *Lactobacillus gasseri* in women [32].

In the present study, we confirmed that, in the obtained endometrium samples, the microbiome was dominated by lactic acid bacilli. It must also be noted that, in the group of patients qualified for the study, we did not confirm the incidence of dysbiosis, which is testified to by the fact that physiological flora constituted 57% of the total endometrium flora, while the presence of pathological microflora was confirmed in a total of 18 patients and, in each patient, lactic acid bacilli were dominant. Thus, it seems that the population of bacteria that may make up both the physiological and pathological microflora may be treated as a physiological component of the entire patient’s endometrial microflora. However, we must not overlook the fact that 33% of the microflora was classified as pathological microflora. In truth, this result could have been a lot lower, as the whole group of *Escherichia coli* bacteria was classified as non-physiological flora; this group includes *E. coli*, as well as *Shigella* and *Salmonella* (10%). The inability to differentiate between individual species within the group is a result of the used sequencing methodology, as well as the genetic similarity of bacteria in the *Enterobacteriaceae* family, which constitutes one of the factors limiting our results. Studies on the microbiome of the vagina and endometrium have confirmed the connection between methods of assisted reproduction [33] and the pathological conditions seen in patients (e.g., chronic inflammation of the mucous membrane of the uterus, endometriosis [34,35], uterine overgrowth, and endometrial cancer [9,11]), which justifies the subject of the undertaken study.

Hashimoto et al., evaluated the effectiveness of IVF in a group of 99 women under the age of 40 years old, as well as the microbiome profile, which is optimal for implantation of the embryo. They did not show significant differences between eubiotic and dysbiotic endometria and the rate of miscarriages and pregnancies. Furthermore, there were no differences confirmed between the microbiome profile of the dysbiotic endometrium of pregnant women and that of women who did not become pregnant [36].

Johnston-MacAnanny et al., confirmed that an inflammatory condition negatively influences reproductive results, while successful treatment is not solely based on antibiotic therapy but may also be combined with modulation of the microflora through the use of prebiotics [37]. In this regard, the studies of Kyono et al., prove to be interesting. They have shown that antibiotic therapy used in patients with dysbiosis may be ineffective, as the lactic acid bacteria may also be targeted by certain antibiotics, especially those with a wide spectrum of activity. Furthermore, the restoration of healthy microflora is dependent on the percentage of bacteria other than *Lactobacillus* and their sensitivity to the antibiotic [38]. The conducted analysis of correlations showed the presence of 12 significant relationships among individual microbes, where 8 of these were almost complete correlations. The obtained results indicated that the microbiome is a dynamic system and that the number of individual kinds or groups of bacteria influences others. This was noted both between individual lactic acid bacilli, where four positive and four negative dependencies were noted, as well as four dependencies between the amount of the physiological and pathological flora. Meanwhile, the occurrence of two negative dependencies seemed to be beneficial, indicating the effectiveness of the therapies, which are targeted at the restoration of the physiological microbiome (*Lactobacillus fermentum* and *Enterococcus faecalis*, r = −0.998903; as well as *Lactobacillus delbrueckii* group and *Escherichia coli* group, r = −0.998903). In the case of the positive relationship between *Lactobacillus FN667084_s* and *Staphylococcus aureus* group (r = 0.997254), as well as that between *Lactobacillus fermentum* and *Streptococcus agalactiae* (r = 0.997254), the data seem to be disconcerting and require further studies, including determining the lifetime of the bacteria in question.

Of course, our study had strengths and limitations.

An undoubted advantage of our research is the fact that, to the best of our knowledge, this is the first study of this type in the Polish population. Microbiome tests are not a standard procedure for diagnosing infertility or monitoring the success of IVF.

It should also be borne in mind that there have been recent legislative changes in Poland, and the in vitro fertilization procedure is not financed/co-financed by government funds [39].

In addition, the obtained results seem very interesting and have been insufficiently described in the literature to date, especially in the context of the positive correlation between *Lactobacillus FN667084_s* and *Staphylococcus aureus* group, as well as that between *Lactobacillus fermentum* and *Streptococcus agalactiae*.

The presented study does have limitations. It must be understood that, by using the NGS method, we were able to detect the nucleotide sequences of microbe DNA, which provided us with knowledge on the subject of taxa and allowed us to characterize the microbiome; however, this is not the same as identifying live bacteria in the studied micro-environment. As such, the results presented here may be treated as a starting point for further analyses, which should concentrate on the connections between microbiome composition and the effectiveness of IVF. Another limitation of the study is the relatively small—but statistically significant—number of patients included in the study and the fact that it was a single-center study. Another important step would include the assessment of the microbiome of the male sperm of infertile couples, in order to better search for connections with infertility. Nevertheless, it should be borne in mind that, in our study, we had clearly defined, strict inclusion and exclusion criteria. One should also not forget about the population and individual variability, which is always important in the context of the analysis of data in the field of microbiology and molecular biology [40,41].

However, taking into account the significance of becoming familiar with the microbiome within the context of the problem of global infertility and the effectiveness of in vitro fertilization methods, the conducted studies were significant, although full conclusions will only be made after a larger number of patients is analyzed and the dependence between the microbiota of the patients qualified for the procedure and the percentage of miscarriages compared with IVF effectiveness is determined.

## 5. Conclusions

While it has not yet been established what is meant by “the ideal composition of the vaginal and endometrial microbiome”, research such as that presented in this paper is an important building block in better understanding the role of microbes in the context of fertility and IVF success. As mentioned earlier, the research presented in this paper can be considered the first stage, and it is possible that, in the future, we will be able to determine whether and how we can influence the creation of beneficial microflora in the context of fertility.

Based on the conducted study, it may be stated that the endometrium is, to a large extent, colonized by lactic acid bacilli. Apart from that, no endometrium dysbiosis was noted in patients qualified for the IVF procedure. The presented study is significant within the context of studies on infertility and the evaluation of IVF effectiveness; however, considering the limitations of the conducted experiment, further studies are justified and necessary.

## Figures and Tables

**Table 1:** Characteristics of women included in the study.

Age (years)	34.09 ± 3.17
Weight (kg)	68.03 ± 14.96
High (cm)	165.36 ± 6.97
BMI	24.99 ± 2.09
Duration of infertility (years)	9.3 ± 2.4
Concentration of beta hCG (IU/L)	21.09 ± 5.89

BMI, body mass index; beta-hCG, beta-human chorionic gonadotropin; average ± standard deviation (x ± SD).

**Table 2:** Inclusion and exclusion criteria for the study.

Inclusion Criteria	Exclusion Criteria
Infertility as defined by Word Health Organization standards	American Fertility Score III/IV and pre-treatment with a gonadotrophin-releasing hormone analog
Written, conscious consent of the patient	No written consent given
Age 18–45	Age < 18 or >45
Qualification and performance of the in vitro procedure	Disqualification of the patient, and excluding her from the IVF procedure in accordance with the criteria and qualifications described in the current recommendations and guidelines
No use of probiotic, prebiotic, or synbiotic preparations for at least 3 months before the examination, regardless of the form of administration	Use of probiotic, prebiotic, or symbiotic preparation for at least 3 months before the examination, regardless of the form of administration
Undergoing sample transfer procedure	
No use of antibiotics in the period of at least 3 months before the examination, regardless of the form of administration	Use of antibiotics in the period of at least 3 months before the examination, regardless of the form of administration
No current or past neoplastic disease	Current or past neoplastic disease
No mental or emotional disorders	Mental or emotional disorders
Caucasian race	Race other than Caucasian
No malformations of the uterus and fallopian tubes	Previous pregnancy or miscarriage in their medical history
No vaginal infections (vaginal discharge, itching, burning, pain, bad smell)	Current or last 3 months before study vaginal infections
No endometriosis	Endometriosis
No use of hormonal contraceptives within 3 months prior to the start of their IVF intake	Used hormonal contraceptives within 3 months prior to the start of their IVF intake

**Table 3:** Vaginal microflora of patients qualified for the study.

Lp.	Strain	Average	95% Confidence Interval	Physiological/Pathological Microflora	% of Total Microflora
1	*Lactobacillus helveticus* group	60,570.66 ± 21,824.62	55,682.2; 65,459.1	Physiological	16
2	*Lactobacillus jensenii* group	42,462.03 ± 38,365.73	28,389.4; 56,534.7	Physiological	11
3	*Lactobacillus gasseri* group	37,687.71 ± 29,282.06	26,947.0; 48,428.5	Physiological	10
4	*Lactobacillus iners*	37,272.93 ± 32,042.41	18,772.2; 55,773.7	Physiological	10
5	*Escherichia coli* group	35,894.00 ± 30,071.02	4336.4; 67,451.6	Pathological	10
6	*Atopobium parvulum*	20,603.00 ± 25,672.43	−43,170.8; 84,376.8	Pathological	6
7	*Bifidobacterium breve*	18,250.67 ± 17,535.96	4771.3; 31,730.0	Physiological and pathological	5
8	*Lactobacillus paracasei* group	17,414.67 ± 25,999.63	−47,172.0; 82,001.3	Physiological	5
9	*Lactobacillus delbrueckii* group	16,862.00 ± 5135.53	4104.6; 29,619.4	Physiological	5
10	*Streptococcus agalactiae*	16,063.33 ± 16,117.75	−23,975.4; 56,102.0	Pathological	4
11	*Staphylococcus aureus* group	14,118.33 ± 24,114.68	−11,188.5; 39,425.1	Pathological	4
12	*Bifidobacterium longum* group	13,680.50 ± 11,412.00	−88,852.2; 116,213.2	Physiological and pathological	4
13	*Gardnerella vaginalis* group	12,532.25 ± 9780.94	4355.2; 20,709.3	Pathological	3
14	*Streptococcus salivarius* group	11,037.50 ± 10,815.23	−6171.9; 28,246.9	Pathological	3
15	*Enterococcus faecalis*	4751.50 ± 6977.68	−6351.6; 15,854.6	Pathological	1
16	*Lactobacillus reuteri* group	3406.22 ± 2057.17	1824.9; 4987.5	Physiological	1
17	*Aerococcus christensenii*	2301.33 ± 1119.73	−480.2; 5082.9	Pathological	1
18	*Lactobacillus fermentum*	1517.33 ± 620.41	−23.8; 3058.5	Physiological	<1
19	*Clostridium fallax*	1032.50 ± 252.44	−1235.6; 3300.6	Physiological	<1
20	*Lactobacillus FN667084_s*	935.67 ± 64.47	775.5; 1095.8	Physiological	<1
21	*Alloscardovia omnicolens*	905.00 ± 69.30	282.4; 1527.6	Pathological with clinical symptoms	<1
22	Lactobacillus_uc	724.00 ± 386.08	−2744.8; 4192.8	Physiological	<1

## Data Availability

The data used to support the findings of this study are included in the article. The data will not be shared because of third-party rights and commercial confidentiality.

## Supplementary Materials

The following supporting information can be downloaded at: https://www.mdpi.com/article/10.3390/jcm11123348/s1. Table S1: Results of post hoc Tukey’s test (*p* < 0.05) presenting statistically significant differences in the number of readings of individual bacterial strains colonizing the endometrium and uterine cervix of patients qualified for the IVF procedure.
