# Supplementary material for: Marking the Profile of the Microflora of the Endometrium and Uterine Cervix in Women as a Potential Factor Determining the Effectiveness of In Vitro Fertilization
Source: J Clin Med. 2022 Jun 10;11(12):3348. doi: 10.3390/jcm11123348 (PMC9224746; doi:10.3390/jcm11123348)
Supplement: Supplementary file 1 [file jcm-11-03348-s001.zip › jcm-1711740-supplementary.pdf]

Table S1. Results of post-hoc Tukey's test ($p < 0.05$) presenting statistically significant differences in the number of readings of individual bacterial strains colonizing the endometrium and uterine cervix of patients qualified for the IVF procedure.

Number	Name of taxon	{1}	{2}	{3}	{4}	{5}	{6}	{7}	{8}	{9}	{10}	{11}	{12}	{13}	{14}	{15}	{16}	{17}	{18}	{19}	{20}	{21}	{22}
**1**	*Aerococcus christensenii*		1.000000	1.000000	0.999999	1.000000	1.000000	1.000000	0.965496	1.000000	1.000000	1.000000	1.000000	0.786143	0.018682	0.866165	0.555109	1.000000	1.000000	1.000000	1.000000	1.000000	1.000000
**2**	*Alloscardovia omnicolens*	1.000000		1.000000	1.000000	1.000000	1.000000	1.000000	0.989252	1.000000	1.000000	1.000000	1.000000	0.937381	0.137542	0.960656	0.823817	1.000000	1.000000	1.000000	1.000000	1.000000	1.000000
**3**	*Atopobium parvulum*	1.000000	1.000000		1.000000	1.000000	1.000000	1.000000	1.000000	1.000000	1.000000	0.999999	0.999998	0.999976	0.507407	0.999993	0.998868	1.000000	0.999995	1.000000	1.000000	1.000000	1.000000
**4**	*Bifidobacterium breve*	0.999999	1.000000	1.000000		1.000000	1.000000	1.000000	0.999635	1.000000	1.000000	0.999997	0.999994	0.925862	0.000569	0.983121	0.632185	1.000000	0.999858	1.000000	1.000000	1.000000	1.000000
**5**	*Bifidobacterium longum* group	1.000000	1.000000	1.000000	1.000000		1.000000	1.000000	0.999987	1.000000	1.000000	1.000000	1.000000	0.999719	0.584255	0.999877	0.996230	1.000000	1.000000	1.000000	1.000000	1.000000	1.000000
**6**	*Clostridium fallax*	1.000000	1.000000	1.000000	1.000000	1.000000		1.000000	0.989717	1.000000	1.000000	1.000000	1.000000	0.939425	0.140186	0.962058	0.827793	1.000000	1.000000	1.000000	1.000000	1.000000	1.000000
**7**	*Enterococcus faecalis*	1.000000	1.000000	1.000000	1.000000	1.000000	1.000000		0.990855	1.000000	1.000000	1.000000	1.000000	0.910382	0.044475	0.950959	0.738653	1.000000	1.000000	1.000000	1.000000	1.000000	1.000000
**8**	*Escherichia coli* group	0.965496	0.989252	1.000000	0.999635	0.999987	0.989717	0.990855		0.987966	0.999989	0.956218	0.948223	1.000000	0.792460	1.000000	1.000000	0.999994	0.701115	0.988563	0.997946	0.999978	0.999881
**9**	*Gardnerella vaginalis* group	1.000000	1.000000	1.000000	1.000000	1.000000	1.000000	1.000000	0.987966		1.000000	1.000000	1.000000	0.647717	0.000131	0.848839	0.292742	1.000000	1.000000	1.000000	1.000000	1.000000	1.000000
**10**	*Lactobacillus delbrueckii* group	1.000000	1.000000	1.000000	1.000000	1.000000	1.000000	1.000000	0.999989	1.000000		1.000000	1.000000	0.999441	0.322463	0.999810	0.990719	1.000000	1.000000	1.000000	1.000000	1.000000	1.000000
**11**	*Lactobacillus fermentum*	1.000000	1.000000	0.999999	0.999997	1.000000	1.000000	1.000000	0.956218	1.000000	1.000000		1.000000	0.752211	0.015366	0.840763	0.514586	1.000000	1.000000	1.000000	1.000000	1.000000	1.000000
**12**	*Lactobacillus FN667084_s*	1.000000	1.000000	0.999998	0.999994	1.000000	1.000000	1.000000	0.948223	1.000000	1.000000	1.000000		0.725658	0.013261	0.820283	0.484761	1.000000	1.000000	1.000000	1.000000	1.000000	1.000000
**13**	*Lactobacillus gasseri* group	0.786143	0.937381	0.999976	0.925862	0.999719	0.939425	0.910382	1.000000	0.647717	0.999441	0.752211	0.725658		0.004716	1.000000	1.000000	0.999626	0.059366	0.934395	0.903806	0.999030	0.996303

**14**	*Lactobacillus helveticus* group	0.018682	0.137542	0.507407	0.000569	0.584255	0.140186	0.044475	0.792460	0.000131	0.322463	0.015366	0.013261	0.004716		0.187162	0.109022	0.347547	0.000015	0.133854	0.003550	0.288012	0.205300
**15**	*Lactobacillus iners*	0.866165	0.960656	0.999993	0.983121	0.999877	0.962058	0.950959	1.000000	0.848839	0.999810	0.840763	0.820283	1.000000	0.187162		1.000000	0.999877	0.207832	0.958599	0.965910	0.999658	0.998551
**16**	*Lactobacillus jensenii* group	0.555109	0.823817	0.998868	0.632185	0.996230	0.827793	0.738653	1.000000	0.292742	0.990719	0.514586	0.484761	1.000000	0.109022	1.000000		0.992921	0.010148	0.818088	0.644217	0.986566	0.967138
**17**	*Lactobacillus paracasei* group	1.000000	1.000000	1.000000	1.000000	1.000000	1.000000	1.000000	0.999994	1.000000	1.000000	1.000000	1.000000	0.999626	0.347547	0.999877	0.992921		1.000000	1.000000	1.000000	1.000000	1.000000
**18**	*Lactobacillus reuteri* group	1.000000	1.000000	0.999995	0.999858	1.000000	1.000000	1.000000	0.701115	1.000000	1.000000	1.000000	1.000000	0.059366	0.000015	0.207832	0.010148	1.000000		1.000000	1.000000	1.000000	1.000000
**19**	Lactobacillus_uc	1.000000	1.000000	1.000000	1.000000	1.000000	1.000000	1.000000	0.988563	1.000000	1.000000	1.000000	1.000000	0.934395	0.133854	0.958599	0.818088	1.000000	1.000000		1.000000	1.000000	1.000000
**20**	*Staphylococcus aureus* group	1.000000	1.000000	1.000000	1.000000	1.000000	1.000000	1.000000	0.997946	1.000000	1.000000	1.000000	1.000000	0.903806	0.003550	0.965910	0.644217	1.000000	1.000000	1.000000		1.000000	1.000000
**21**	*Streptococcus agalactiae*	1.000000	1.000000	1.000000	1.000000	1.000000	1.000000	1.000000	0.999978	1.000000	1.000000	1.000000	1.000000	0.999030	0.288012	0.999658	0.986566	1.000000	1.000000	1.000000	1.000000		1.000000
**22**	*Streptococcus salivarius* group	1.000000	1.000000	1.000000	1.000000	1.000000	1.000000	1.000000	0.999881	1.000000	1.000000	1.000000	1.000000	0.996303	0.205300	0.998551	0.967138	1.000000	1.000000	1.000000	1.000000	1.000000	
